# Positive Effect of Super-Resolved Structural Magnetic Resonance Imaging for Mild Cognitive Impairment Detection

**DOI:** 10.3390/brainsci14040381

**Published:** 2024-04-14

**Authors:** Ovidijus Grigas, Robertas Damaševičius, Rytis Maskeliūnas

**Affiliations:** 1 Faculty of Informatics, Kaunas University of Technology, 50254 Kaunas, Lithuania; o.grigas@ktu.edu (O.G.); rytis.maskeliunas@ktu.lt (R.M.); 2 Faculty of Applied Mathematics, Silesian University of Technology, 44-100 Gliwice, Poland

**Keywords:** magneticresonance imaging, super-resolution, mild cognitive impairment, hyperparameter optimization, Pareto optimality, Markov blanket

## Abstract

This paper presents a novel approach to improving the detection of mild cognitive impairment (MCI) through the use of super-resolved structural magnetic resonance imaging (MRI) and optimized deep learning models. The study introduces enhancements to the perceptual quality of super-resolved 2D structural MRI images using advanced loss functions, modifications to the upscaler part of the generator, and experiments with various discriminators within a generative adversarial training setting. It empirically demonstrates the effectiveness of super-resolution in the MCI detection task, showcasing performance improvements across different state-of-the-art classification models. The paper also addresses the challenge of accurately capturing perceptual image quality, particularly when images contain checkerboard artifacts, and proposes a methodology that incorporates hyperparameter optimization through a Pareto optimal Markov blanket (POMB). This approach systematically explores the hyperparameter space, focusing on reducing overfitting and enhancing model generalizability. The research findings contribute to the field by demonstrating that super-resolution can significantly improve the quality of MRI images for MCI detection, highlighting the importance of choosing an adequate discriminator and the potential of super-resolution as a preprocessing step to boost classification model performance.

## 1. Introduction

Mild cognitive impairment (MCI) is considered as a prodromal stage of Alzheimer’s disease based on clinical symptoms [1]. It is also a transitional period between healthy aging, where cognitive decline is a normal phenomena, and dementia [2]. MCI usually impacts cognitive abilities such as reasoning, memory, and logic [3]. People with this condition are usually forgetful, and need more time to think or express certain thoughts. However, they do not need assisted living facilities, because they are able to take care of themselves in everyday life. People with MCI may or may not convert to Alzheimer’s disease [4,5,6] or dementia [4]. The condition every year affects millions of people worldwide and attracts large investments from governments into research and drug production. There is no cure for this disease; however, certain treatments can reduce symptoms if applied on time. Therefore, early diagnosis is crucial, which allows patients and their caregivers enough time to prepare for the future. However, currently, there is no standardized assessment that would allow one to accurately diagnose MCI [7]. Due to this fact, researchers try to find new ways to accurately detect MCI via a vast number of different data modalities, for example, electroencephalogram (EEG) [8], 18F fluoro-deoxy-glucose positron emission tomography (FDG-PET) [9], cerebrospinal fluid (CSF) biomarkers [10], natural language [11], or T1w and T2w MRI [12,13]. Neuroimaging markers are becoming more popular and show great potential towards accurately identifying MCI [14,15]. Certain structural changes in the brain are present when a patient has MCI, for example, a decrease in gray matter volume in the medial temporal lobe [16] and hippocampal, entorhinal cortex atrophy with cortical volume decrease [17,18]. The task of detecting MCI is challenging, because it usually affects elderly people, and it is hard to distinguish if changes in the brain volume are impacted due to normal aging [19] or due to MCI, since some of the regions, for example, the temporal lobe, show a volume decrease in both scenarios. Therefore, it is crucial for the tools to not only focus on the specific known regions of interest (ROI), but also to incorporate other regions of the brain, which may have a correlation to the presence of MCI. Particularly, enhancing smaller regions with finer details in MRI may allow diagnostic tools such as deep learning (DL) models to find other important regions and more accurately detect MCI.

Super-resolution technology has been a helpful tool in many different science areas, for example, hyperspectral imaging [20], nature sciences [21], satellite imagery [22], license plate recognition [23], and medical imaging—this paper. This technology utilizes deep learning models to increase the quality of low-resolution data by upscaling and reconstructing an image, which would be accurate and meaningful. Usually, researchers focus their super-resolution solutions into improvements in a controlled environment, where a small dataset with a highly specialized solution can reach high results, but all of these solutions are impractical in real world scenarios, where data are usually not a controlled factor. A small change in the data domain means the model will be incapable of reconstructing that image. In these challenging scenarios, “real-world” super-resolution solutions become useful. These solutions do not rely on paired image datasets, where a low-resolution image is known for each high-resolution image. Here, low-resolution images are generated randomly by utilizing degradation (augmentation) techniques in a completely random order [24]. By using degradation techniques, we can cover a wider distribution of possible input images, making the model more practical. Therefore, this paper utilizes the real-world super-resolution paradigm. Another problem with super-resolution is that many solutions are not focusing on the perceptual quality of the reconstructed images. Many researchers only focus on peak signal-to-noise ratio (PSNR) and structural similarity index measure (SSIM) to report their results, even though subjectively generated images are blurry and noisy. In the medical imaging field, preserving the structural part of the image quality is as important as the perceptual part. Therefore, just like in our previous paper [25], we maintain the focus to improve the main important aspects of the image quality—structural and perceptual.

Deep learning model hyperparameter optimization plays a crucial role in enhancing the performance and accuracy of diagnostic models in the field of medical imaging [26]. By fine-tuning parameters such as learning rates, layer configurations, and activation functions, these models can be better adapted to the nuances of medical datasets, which often contain complex patterns and subtle features critical for accurate diagnosis [27]. Optimizing hyperparameters enables the models to effectively learn from high-dimensional imaging data, such as MRI, CT scans, and X-rays, leading to improved sensitivity and specificity in detecting and classifying diseases [28].

In medical imaging diagnostics, the stakes are high, as the early and accurate identification of conditions can significantly impact patient outcomes [26]. Hyperparameter optimization ensures that deep learning models are not only tailored to the unique challenges of medical data but also generalized enough to handle variations across different imaging modalities and patient demographics [27]. This process also helps in reducing overfitting, ensuring that the model’s performance is robust across unseen data, which is paramount in clinical settings where the model’s predictions can directly influence treatment decisions [29].

Bayesian networks, a class of probabilistic graphical models, represent complex relationships between a set of variables using directed acyclic graphs (DAGs) [30]. Each node in a Bayesian network symbolizes a variable, while the edges denote conditional dependencies between them, encapsulating the probabilistic influences of variables on one another [31]. In the context of hyperparameter optimization for machine learning models, Bayesian networks serve as a powerful tool to model and understand the intricate dependencies between various hyperparameters and their impact on model performance metrics [32]. By capturing these relationships, Bayesian networks facilitate a structured exploration of the hyperparameter space, enabling the identification of optimal configurations [33]. This approach not only streamlines the optimization process by focusing on the most influential hyperparameters but also enhances the efficiency and efficacy of the model tuning phase, leveraging probabilistic reasoning to guide the search towards hyperparameter sets that are likely to yield improved performance outcomes [32,33].

The novelty and contribution of this study lie in its innovative integration of super-resolution imaging techniques and advanced machine learning optimization strategies to enhance the detection of MCI from structural MRI scans. Specifically, the study introduces the following novel contributions to the field of medical imaging and diagnostics:

- By employing super-resolution techniques within a generative adversarial network (GAN) framework, this study improves the perceptual quality of structural MRI images. This enhancement is pivotal, as higher-resolution images can reveal subtle brain changes associated with MCI, which are often not discernible in low-resolution scans.
- This research advances the state of the art by incorporating a combination of loss functions, including perceptual loss and adversarial loss, to not only increase the resolution of MRI images but also to maintain their diagnostic integrity. This approach addresses common issues in super-resolution, such as checkerboard artifacts, ensuring that the enhanced images are both high in quality and clinically reliable.
- A key contribution is the application of a POMB approach for hyperparameter optimization in deep learning models used for MCI detection. This method systematically evaluates and selects hyperparameters to balance model complexity and performance, reducing overfitting and improving generalizability. The use of POMB in this context is novel, offering a structured framework for enhancing model accuracy in medical diagnostics.
- This study validates the effectiveness of super-resolution preprocessing on MCI detection across various state-of-the-art deep learning architectures. This empirical evidence supports the premise that super-resolution can serve as a valuable preprocessing step in medical imaging analysis, potentially applicable beyond MCI detection.
- The investigation into the impact of different discriminator architectures within the GAN framework on the quality of super-resolved images underscores the critical role of discriminator choice. This insight contributes to the broader understanding of how GAN components influence the outcome of super-resolution tasks, guiding future research and application in neuroimaging enhancement.

The main purpose of this study is to improve the processing of MRI data and validate the proposed methodology effectiveness in mild cognitive impairment detection.

The rest of the paper is organized as follows: Section 2 discusses the related studies. Section 3 explains the proposed methodology improvements to our previous work to improve perceptual quality of MR images. Section 4 presents the research findings in terms of quantitative and qualitative evaluation of the proposed methodology. Section 5 discusses and summarizes the findings and presents the conclusions.

## 2. Related Works

Neuroimage enhancement is a compelling field of study that is increasingly gaining traction in research circles. As advancements in imaging technology continue to improve, the need for enhancing neuroimages to extract more accurate diagnostic information becomes more pronounced. For identification of similar studies, we utilized the database engines—Web of Science, Scopus, IEEE Xplore, Springer Link, and Science Direct (Last accessed on 7 March 2024). We constructed the search queries using these keywords: super, resol*, mild*, mci, detect*, class*. We combined the keywords with Boolean operators (AND, OR) and filtered only to articles and conference proceedings. Asterisk (*) was used to include words with different suffixes. Only sources published after 2014 and written in English were included. After the initial screening, 157 sources were identified. After removing duplicates, 86 entries were left. After the title and abstract screening, 22 sources were left. After full-text eligibility review, 6 sources were included in the study, and are compared in Table 1.

Alwakid et al. [34] used ESRGAN  [35] to upscale retinal images, and then used the Inception v3 model [36] to classify the images into five different classes of diabetic retinopathy (mild, moderate, proliferative, severe, undetected). The dataset they used was APTOS [37]. Their experiments show that using super-resolution improves baseline accuracy by nearly 18%.

Tan et al. [38] used the SRGAN [39] model to upscale computed tomography (CT) scans of patient lungs, which then were used to classify with the VGG-16 [40] model whether the patient has COVID-19 pneumonia or not. The dataset they used was COVID-CT [41]. Their experiments also show that the super-resolution technique improves baseline accuracy by approximately 8%.

Nagayama et al. [42] utilized super-resolution software PIQE (SR-DLR) [43], which is being sold by Canon alongside their CT scanners. It is a custom 3D CNN trained on CT images. No other details are disclosed by the company. However, validation of the method shows that it improves not only image quality, but also the detection of coronary lumens, calcifications, and non-calcified plaques approximately. The methodology of the source describes using the detectability index to measure performance [44]. The authors have not disclosed the dataset used in their study. The method shows an approximately 5% improvement over the other state-of-the-art solutions.

De Farias et al. [45] slightly modified GAN-CIRCLE [46] and used it to evaluate whether super-resolution improves feature selection in CT scans. For this reason, they used principal component analysis (PCA) with spatial pyramid pooling (SPP), and then checked which features were selected as the most important ones. The authors used the NSCLC [47] dataset. Experiments show that using super-resolution improves feature selection by relatively 2% if ranking by the feature importance using the intraclass correlation coefficient (ICC).

Huang et al. [48] combined wavelet transform with DDGAN [49] to improve the resolution of the ADNI [50] dataset images. They used T1w image slices from the coronal plane and performed ×4 times upscaling from 48 × 48 to 192 × 192 resolution. First, they downscaled the original images and then tried to reconstruct them with super-resolution. The experiments with the support vector machine (SVM) as classifier show a relative 2% performance increase by using super-resolution.

Zhang et al. [51] used a custom 3D encoder–decoder GAN with residual connections to super-resolve T2w MRI images. The dataset that they used consisted of 200 patients who went through an inflammatory bowel disease clinical trial, but it is not publicly available. After super-resolving the images, they used ResNet to classify the images, and found no improvement over the baseline.

**Table 1:** Comparison of different approaches for image super-resolution and classification in medical imaging.

Reference	Super-Resolution Model	Classification Model	Dataset	Improvement
*Fundus photography*				
Alwakid et al. [34]	ESRGAN	Inception v3	APTOS	18%
*CT Scans*				
Tan et al. [38]	SRGAN	VGG-16	COVID-CT	8%
Nagayama et al. [42]	PIQE (SR-DLR)	-	-	5%
de Farias et al. [45]	Modified GAN-CIRCLE	PCA+SPP	NSCLC	2%
*MRI*				
Huang et al. [48]	DDGAN	SVM	ADNI	2%
Zhang et al. [51]	3D Encoder–Decoder GAN	ResNet	-	0%
This paper	Hybrid Transformer GAN	Various Models	ADNI, OASIS-4	1–4%

Naturally, the accuracy varies depending on the application and the size of the dataset used in training, but overall, super-resolution technology improves the accuracy of classification models in the majority of tasks.

## 3. Materials and Methods

### 3.1. Experimental Data

For the super-resolution model improvements, we used the same ultra-high-resolution MRI dataset “human phantom” [52] that we used in our previous work [25]. (Dataset available online: https://datadryad.org/stash/dataset/doi:10.5061/dryad.38s74—accessed on 5 March 2024). All of the preprocessing steps were also unchanged.

A short description of both datasets is available in Table 2. More details of how the data were prepared are available in Section 4.1.

### 3.2. Improvement of Super-Resolution Hybrid Transformer GAN

The baseline of the improvements for this study is our previously published method [25], which increases the resolution of structural MRIs while preserving perceptional image quality. It uses hybrid attention transformer (HAT) as a generator and introduces an adversarial training pipeline, which allows one to super-resolve structural MRI and decrease its blurriness and noise. In this study, we employ the following improvements over the previous method: (1) a deeper/denser network for discriminator of hybrid attention transformer (HAT) model generator, (2) use of Wasserstein GAN (WGAN) loss and frequency domain loss, (3) addition of more augmentation techniques, (4) modification of upsampling layer of generator model, and (5) implementation of hyperparameter optimization using POMB.

#### 3.2.1. Usage of Deeper/Denser Network for the Discriminator

To use the deeper model for discriminator, we experimented with various existing model architectures, which are briefly described in Table 3.

#### 3.2.2. Definition of Loss Function

One of the improvements proposed by our previous work was the use of Wasserstein GAN [57] for adversarial training. WGAN proved to make the training of models more stable. Therefore, we replaced vanilla GAN loss with WGAN loss. WGAN loss is defined as in Equations (1) and (2):

(1) $$L_{G}=\bar{G(z)},$$

(2) $$L_{D}=\bar{x}-\bar{G(z)},$$

where *z* is a fake image and *x* is a target image. WGAN discriminator is simply called “critic”, because it is only yielding a score of the generated image. The score itself is just a mean value of the tensor.

The next change to our methodology was to swap perceptual-style reconstruction loss with LPIPS loss. It forces generator to focus a bit more on the contents/features of the generated images, rather than on the style, since the loss combines features from multiple layers in the network. The loss is just a LPIPS metric defined in Equation (25) calculation on which gradient descent can then be used.

For pixel-level loss, we used Charbonnier loss for the same reasons that it is a better variant of mean absolute error (MAE) loss, and it is proven to make training more stable and make models produce images with better visual results [58,59,60]. Charbonnier loss is defined in Equation (3).

(3) $$L_{Charbonnier}=\frac{\sum_{i=1}^{n}\sqrt{{\left(y_{i}-x_{i}\right)}^{2}+\epsilon^{2}}}{n},$$

The last change was to introduce frequency domain-based loss function, which uses Fast Fourier Transform (FFT). FFT is widely used algorithm in many different science fields. It is usually used to reduce noise in images by transforming images from spacial to frequency domain and applying filters [61] to the extracted frequencies. The main idea of frequency domain loss is comparing images pixel-wise like one could do in spacial domain with L1 or L2 loss, but doing so in frequency domain makes the loss slightly more sensitive to blurriness and noise, helps in preserving high-frequency features in images, and overall yields better perceptual quality [62,63,64]. Loss equation is defined in Equation (6), which is an L1 loss between amplitudes and phases of two distinct images.

(4) $$A_{x_{i}},P_{x_{i}}=FFT\left(x_{i}\right),$$

(5) $$A_{y_{i}},P_{y_{i}}=FFT\left(y_{i}\right),$$

(6) $$L_{FD}=\frac{1}{n}\sum_{i=1}^{n}\left(∥A_{x_{i}}-A_{y_{i}}∥+∥P_{x_{i}}-P_{y_{i}}∥\right),$$

where *x* is a high-resolution image, *y* is a generated image, and FFT is a fast Fourier transform applied to 2D image, *n* is a number of samples in the mini-batch and *i* is the index of the sample in the mini-batch.

Combined loss for generator is defined in Equation (7). For discriminator, we used defined discriminator adversarial loss Equation (2).

(7) $$L=L_{Charbonnier}+L_{FD}+L_{G}+L_{LPIPS}$$

#### 3.2.3. Image Augmentation Techniques

Our previous work was following [65]’s described augmentation pipeline, which was developed to train the models to be more generic due to the fact that the training is based on applying various degradation functions to the high-quality images, instead of using paired high-/low-quality images for direct input to the model. The use of randomness in the degradation pipeline trains the model to be more stable given various unknown levels of blurriness, noise, etc., in low-quality images. This branch of super-resolution research is called “real-world” super-resolution. Usually, researchers avoid it because the model performance will be lower than the model trained on paired image dataset. This happens because in controlled environments, models can learn the training set image distribution quite well, but once the low-quality input image is not entirely lying within training set image distribution, generated results will be low-quality.

**Figure 1:**
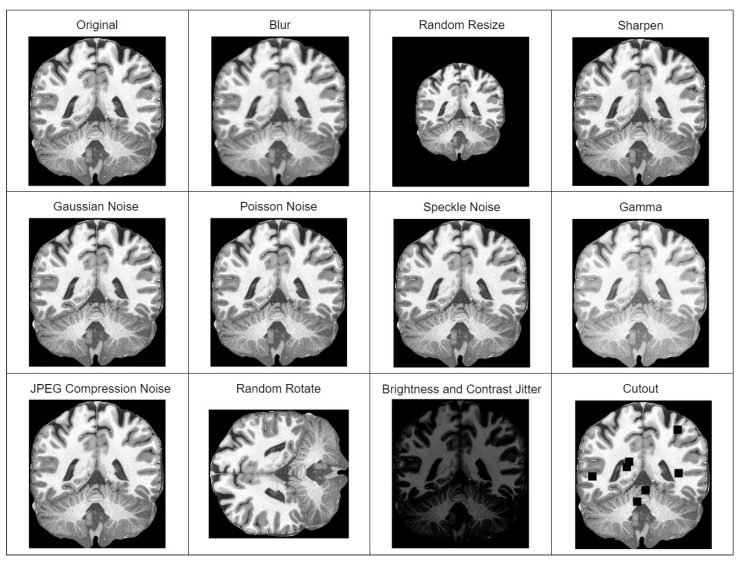
Image augmentations (degradations) used in the training of super-resolution model. Different degradation method outputs are applied to a single extracted slice of T1w MRI of a healthy Caucasian male from “human phantom” dataset [52].

In our case, a model used for sMRI super-resolution must be practical and capable of dealing with a wider distribution of input images than the training set. Hence, the extensive application of random augmentations (degradations) during training. Original pipeline includes blur, resize, Gaussian noise, Poisson noise, speckle noise, and jpeg compression noise transformations applied in random sequence multiple times. We extended the original pipeline with the additional random augmentations of brightness and contrast jitter, sharpening, gamma, cutout, and random rotation transformations. All used augmentations are depicted in Figure 1.

#### 3.2.4. Modified Upsampling Layer of Generator Model

In our methodology, we use HAT generator [66]. Originally, it uses so called “pixel-shuffle” for the upsampling of the tensors, as described in [67]. But this technique is known for being used in classical super-resolution tasks, where perceptual quality is not the main selling point. For real-world super-resolution tasks, the typically used upsampling technique is called “nearest+conv”, which uses deconvolution with overlapping to reduce “checkerboard” artifacts in generated images [68].

### 3.3. Hyperparameter Optimization Using Pareto Optimal Markov Blanket

#### 3.3.1. Types of Hyperparameters

Deep learning model architecture hyperparameters can be intricately described and optimized using the framework of Bayesian networks. This approach uses probabilistic graphical models to represent the conditional dependencies between hyperparameters and the performance metric(s) of interest, enabling systematic exploration and understanding of the hyperparameter space. Four types of hyperparameters are possible in a Bayesian network of hyperparameters:

- A hyperparameter $X_{i}$ is conditionally independent of the hyperparameter $Y_{i}$ given *S* if and only if $P\left(X_{i}|Y_{i},S\right)=P\left(X_{i}|S\right)$.
- A hyperparameter $X_{i}\in R$ is strongly relevant to the target variable *T* if and only if $\forall S\subseteq R\setminus \{X_{i}\}$, s.t. $P\left(X_{i}|S\right)\neq P\left(X_{i}|S,T\right)$.
- A hyperparameter $X_{i}\in R$ is irrelevant to a target variable *T* if and only if $\forall S\subseteq R\setminus \{X_{i}\}$, s.t. $P\left(X_{i}|S,T\right)=P\left(S|T\right)$.
- A hyperparameter $X_{i}$ is redundant for the target variable *T* if and only if it is weakly relevant to target variable *T* and has a Markov blanket, $MB(X_{i})$, then it is a subset of the Markov blanket of $MB_{T}$.

The categorization of hyperparameters as conditionally independent, strongly relevant, irrelevant, and redundant critically informs their inclusion or exclusion for hyperparameter optimization. Conditionally independent hyperparameters are optimized separately; strongly relevant ones are essential and included for optimal performance, while irrelevant and redundant hyperparameters are excluded to streamline the optimization process and avoid overfitting. This selection strategy allows us to achieve an efficient balance between maximizing model performance and maintaining a concise set of hyperparameters, facilitating a targeted and effective tuning process.

#### 3.3.2. Bayesian Network of Hyperparameters

A Bayesian network for the optimization of the hyperparameters of a deep learning model can be represented as a directed acyclic graph (DAG) G=(V,E), where *V* is the set of nodes and *E* is the set of directed edges between these nodes.

Let $H=\{h_{1},h_{2},\ldots ,h_{n}\}$ be the set of hyperparameters of the deep learning model, such as the learning rate, the number of layers, the number of neurons per layer, the type of activation function, and the dropout rate, where each $h_{i}$ is a hyperparameter subject to optimization.

Let $M=\{m_{1},m_{2},\ldots ,m_{k}\}$ represent the set of performance metrics, which are the results measured to evaluate the performance of the model under the configuration defined by *H*. The optimization process seeks to find an optimal configuration $H^{*}=\left\{h_{1}^{*},h_{2}^{*},\ldots ,h_{n}^{*}\right\}$ such that the performance metrics in *M* are optimized (maximized or minimized) according to the specified goals of the model.

Directed edges between nodes signify conditional dependencies. For example, if the performance metric node $m_{i}$ (e.g., validation accuracy) is conditionally dependent on the hyperparameters’ nodes *H*, then there exists a directed edge from each $h_{j}\in H$ to $m_{i}$.

Strongly relevant hyperparameters are directly linked to the performance metrics nodes with directed edges, indicating a direct influence on the model’s output. The network highlights these hyperparameters as critical nodes whose values significantly affect the target metrics, necessitating careful optimization.

The Bayesian network helps with conditional independence through the absence of direct paths between certain hyperparameter nodes when conditioned on other nodes. For example, if the hyperparameter *X* is conditionally independent of *Y* given *Z*, the network will not have a direct edge from *X* to *Y* when *Z* is present, highlighting that *X*’s effect on *Y* is mediated through *Z*.

Irrelevant hyperparameters do not have direct or indirect paths to the performance metrics nodes, indicating their lack of influence on the model’s outcomes. In the Bayesian network, these hyperparameters might be isolated or only connected to other irrelevant hyperparameters, serving as a visual cue for potential exclusion from the optimization process to simplify the model and reduce computational complexity.

Redundant hyperparameters are represented in the network by their connections to the same performance metrics or strongly relevant hyperparameters as other nodes, indicating overlapping influences. Redundant hyperparameters might form clusters within the network, suggesting areas where simplification could occur without loss of predictive power, as their removal or consolidation can lead to a more streamlined and efficient optimization process.

### 3.4. Conditional Probability Table

Each node $v_{i}\in V$ is associated with a probability distribution that quantifies the uncertainty about its values. The conditional probability table (CPT) for a performance metric node $m_{i}$, given hyperparameters *H*, quantifies how hyperparameters influence performance metrics, and can be formally defined as $P(m_{i}|H)$. For instance, the CPT for the performance metric node quantifying accuracy of classification can be represented as

(8) $$P(\mathrm{Accuracy}|h_{1},h_{2},\ldots ,h_{n})=p,$$

where *p* is the probability of achieving a certain level of accuracy given specific values of the hyperparameters $h_{1},h_{2},\ldots ,h_{n}$.

CPTs provide the quantitative backbone of a Bayesian network, specifying the probabilities of a node given its parents, thereby encapsulating the strength and nature of the dependencies among variables.

#### 3.4.1. Faithfulness of Bayesian Network

Further, we introduce the faithfulness assumption that asserts that all and only the conditional independencies observed in the data are reflected in the network’s structure, meaning that the network’s edges (or lack thereof) and the CPTs together accurately model the true underlying probabilistic relationships among the variables, which implies that for a Bayesian network to be faithful to its represented domain, its CPTs must not only be consistent with the observed data but also align with the network’s structure in portraying the correct dependencies and independencies.

Assume that *G* denotes a Bayesian network, and *P* represents a joint probability distribution through the set of hyperparameters R. So, *G* is faithful to *P* if *P* captures all and only the conditional independencies among the hyperparameters in *G*. The faithfulness condition, a critical assumption in the construction of Bayesian networks, stipulates that all observed conditional independencies in the data are accurately reflected in the network structure. This condition directly impacts the assessment of conditional dependencies among hyperparameters and performance metrics, ensuring that the relationships modeled in the Bayesian network truly represent the underlying data generation process. When identifying the POMB, the faithfulness condition guarantees that the dependencies and independencies inferred from the network are reliable, thereby enabling a more accurate selection of hyperparameters that are genuinely predictive of model performance without being redundant. By adhering to the faithfulness condition, the process of deriving the POMB becomes more robust and grounded in the actual interactions between hyperparameters and outcomes, leading to an optimization strategy that is both effective and reflective of true data-driven insights.

#### 3.4.2. Pareto Optimal Markov Blanket (POMB)

Before defining the Pareto optimal Markov blanket (POMB), we introduce some necessary concepts:

The Markov blanket of a target variable *T*, denoted as MB(T), is the minimal subset of hyperparameters in a dataset *D* such that *T* is conditionally independent of D∖MB(T) given MB(T). Formally, for any hyperparameter X∈D∖MB(T),

(9) P(T|MB(T),X)=P(T|MB(T)).

A hyperparameter set *S* is Pareto optimal if there exists no other hyperparameter set $S^{\prime }$ such that $S^{\prime }$ is strictly better than *S* in at least one criterion (e.g., relevance to *T*) without being worse in another (e.g., redundancy).

Now, we are ready to define a Pareto optimal Markov blanket: A Markov blanket MB(T) is Pareto optimal if for every hyperparameter X∈MB(T) and any potential hyperparameter Y∉MB(T), adding *Y* to or removing *X* from MB(T) cannot make MB(T) more predictive of *T* without increasing the redundancy among the hyperparameters in MB(T). Formally, MB(T) is Pareto optimal if for any X∈MB(T) and any Y∉MB(T),

(10) $$∄\;MB^{\prime }\left(T\right):\left(\mathrm{Pred}\left(MB^{\prime }\left(T\right),T\right)>\mathrm{Pred}\left(MB\left(T\right),T\right)\right)\wedge \left(\mathrm{Red}\left(MB^{\prime }\left(T\right)\right)\leq \mathrm{Red}\left(MB\left(T\right)\right)\right),$$

where Pred(MB,T) measures how well MB predicts *T*, and Red(MB) quantifies the redundancy within the hyperparameters in MB.

The evaluation process can be formalized using a multi-objective optimization framework, where we define two objective functions: one for predictive performance ($f_{\mathrm{Pred}}$) and another for redundancy ($f_{\mathrm{Red}}$). The goal is to maximize predictive performance while minimizing redundancy.

#### 3.4.3. Pareto Optimality

Given a Markov blanket MB(T) for a target variable *T*, we define the following optimization problem:

(11) $$\begin{array}{cc} \max & \;f_{\mathrm{perf}}\left(MB\left(T\right)\right) \end{array}$$

(12) $$\begin{array}{cc} \min & \;f_{\mathrm{red}}\left(MB\left(T\right)\right) \end{array}$$

subject to MB(T)⊆H, where H is the set of all possible hyperparameters.

$f_{\mathrm{perf}}\left(MB\left(T\right)\right)$ is the predictive performance metric, which could be precision, F1 score, or any other relevant performance metric; and $f_{\mathrm{red}}\left(MB\left(T\right)\right)$ quantifies the redundancy within the Markov blanket, possibly measured by mutual information or correlation among hyperparameters in MB(T).

Pareto optimality comes into play when selecting the optimal MB(T), where a solution $MB^{*}\left(T\right)$ is Pareto optimal if there does not exist another MB(T) such that

(13) $$\begin{array}{cc} f_{\mathrm{perf}}\left(MB\left(T\right)\right) & >f_{\mathrm{perf}}\left(MB^{*}\left(T\right)\right) \end{array}$$

(14) $$\begin{array}{cc} f_{\mathrm{red}}\left(MB\left(T\right)\right) & <f_{\mathrm{red}}\left(MB^{*}\left(T\right)\right) \end{array}$$

without worsening the other objective. The collection of all Pareto optimal solutions constitutes the Pareto front, from which the optimal Markov blanket can be selected according to specific criteria or preferences.

#### 3.4.4. Ranking Markov Blankets

Ranking Markov blankets by Pareto optimality criteria within a hyperparameter optimization context involves evaluating each Markov blanket according to multiple objectives, aiming to maximize predictive performance while minimizing redundancy. This approach is rooted in multi-objective optimization, where Pareto optimality provides a framework to navigate trade-offs between competing objectives.

A Markov blanket $MB_{1}$ is said to Pareto dominate another $MB_{2}$ if and only if $MB_{1}$ is not worse than $MB_{2}$ in all objectives and strictly better in at least one objective. Formally, given two objectives—predictive performance ($f_{\mathrm{perf}}$) and redundancy ($f_{\mathrm{red}}$)—$MB_{1}$ dominates $MB_{2}$ if $f_{\mathrm{perf}}\left(MB_{1}\right)\geq f_{\mathrm{perf}}\left(MB_{2}\right)$ (higher is better for performance) $f_{\mathrm{red}}\left(MB_{1}\right)\leq f_{\mathrm{red}}\left(MB_{2}\right)$ (lower is better for redundancy) At least one of these inequalities is strict.

The Pareto front consists of all non-dominated Markov blankets. These are the MBs for which no other MB exists that Pareto dominates. The Pareto front represents the set of optimal trade-offs between the objectives, where no single MB is universally best, but each is optimal within the context of a specific balance between performance and redundancy.

Ranking Markov blankets (MBs) by Pareto optimality criteria involves a systematic process that can be detailed as follows:

The Pareto front, PF, is made up of non-dominated MBs. An MB, $MB_{i}$, is considered non-dominated if there is no other $MB_{j}$ such that

(15) $$f_{\mathrm{perf}}\left(MB_{j}\right)\geq f_{\mathrm{perf}}\left(MB_{i}\right)\;\mathrm{and}\;f_{\mathrm{red}}\left(MB_{j}\right)\leq f_{\mathrm{red}}\left(MB_{i}\right),$$

with at least one inequality being strict. Here, $f_{\mathrm{perf}}$ and $f_{\mathrm{red}}$ denote the performance and redundancy metrics, respectively.

Within PF, MBs can be further ranked based on secondary criteria. Let $D(MB_{i})$ represent the degree of dominance of $MB_{i}$, defined as the number of MBs that $MB_{i}$ dominates. The secondary ranking can then consider $D(MB_{i})$, specific preferences, or additional metrics:

(16) $$\mathrm{Rank}\left(MB_{i}\right)=g\left(D(MB_{i}),\mathrm{Preferences},\mathrm{Additional}\;\mathrm{Metrics}\right),$$

where *g* is a function that combines these factors into a comprehensive ranking.

The crowding distance, $CD_{i}$, for a MB in a dense region of PF, is used to prefer solutions with a broader spread of trade-offs:

(17) $$CD_{i}=\sum_{k=1}^{K}\left(f_{k}^{\mathrm{next}}\left(MB_{i}\right)-f_{k}^{\mathrm{prev}}\left(MB_{i}\right)\right),$$

where *K* is the number of objectives, and $f_{k}^{\mathrm{next}}$ and $f_{k}^{\mathrm{prev}}$ are the values of the *k*-th objective for the next and previous MBs in the ranking, respectively.

The ranking of MBs can be dynamically updated as new data or insights become available. Let ${\mathrm{PF}}_{\mathrm{new}}$ represent the updated Pareto front, then

(18) $${\mathrm{PF}}_{\mathrm{new}}=\mathrm{Update}\left(\mathrm{PF},\mathrm{New}\;\mathrm{Data}\right),$$

where Update(·) is a function that integrates new candidates into PF and removes dominated ones.

This approach detailed in Algorithm 1 provides a comprehensive framework for ranking MBs in the context of Pareto optimality, balancing between performance optimization and redundancy minimization.

Ranking by Pareto optimality criteria thus involves not only identifying the set of optimal compromises between competing objectives, but also refining within this set based on broader considerations of diversity, dominance, and specific preferences, which ensures a comprehensive exploration of the hyperparameter space, guiding the selection towards solutions that best balance the inherent trade-offs in model optimization.

#### 3.4.5. POMB Construction Criteria

In addition, we introduce two criteria, V-structures and D-separation, which are used to construct the POMB.

In a faithful Bayesian network, an MB of the target variable *T*, $MB_{T}$, in a set R is an optimal set of hyperparameters, composed of parents, children, and spouses. All other hyperparameters are not conditionally dependent on the target variable *T* given $MB_{T}$, $\forall X_{i}\in R\setminus \left(MB_{T}\cup T\right)$, s.t. $X_{i}⊥T|MB_{T}$.

A V-structure in a Bayesian network occurs when two nodes (hyperparameters) have arrows pointing to a common child, but there is no direct edge between the two parent nodes. This structure is crucial for understanding conditional independence and dependence relationships because it can introduce conditional dependencies that are not apparent through direct connections alone. If there is no arrow between hyperparameter $X_{i}$ and hyperparameter $Y_{i}$, and hyperparameter $Z_{i}$ has two incoming arrows from $X_{i}$ and $Y_{i}$, respectively, then $X_{i}$, $Z_{i}$, and $Y_{i}$ form a V-structure $X_{i}\to Z_{i}\leftarrow Y_{i}$. In the context of a POMB, V-structures can influence the determination of which hyperparameters are part of the Markov blanket. Specifically, the spouse (SP) components of a Markov blanket are identified through V-structures, where the spouses are the other parents of the target variable’s children. Understanding and identifying V-structures help in correctly identifying these spouses, ensuring the Markov blanket is accurately defined, which is a step toward achieving Pareto optimality by considering redundancy and relevance of hyperparameters.
**Algorithm 1** Ranking Markov blankets by Pareto optimality criteria 1:**Input:** Set of Markov blankets MBs, performance function $f_{\mathrm{perf}}$, redundancy function $f_{\mathrm{red}}$ 2:**Output:** Ranked list of Markov blankets $MBs_{\mathrm{ranked}}$ 3:**procedure** IdentifyParetoFront(MBs) 4:    Initialize ParetoFront←∅ 5:    **for** each $MB_{i}$ in MBs **do** 6:        Dominated ← False 7:        **for** each $MB_{j}$ in MBs **do** 8:           **if** $MB_{j}$ Pareto dominates $MB_{i}$ **then** 9:               Dominated ← True10:               **break**11:           **end if**12:        **end for**13:        **if** not Dominated **then**14:           Add $MB_{i}$ to ParetoFront15:        **end if**16:    **end for**17:    **return** ParetoFront18:**end procedure**19:**procedure** SecondaryRanking(ParetoFront)20:    Rank ParetoFront based on secondary criteria (degree of dominance, preferences, etc.)21:**end procedure**22:**procedure** ApplyCrowdingDistance(ParetoFront)23:    Calculate crowding distance for each MB in ParetoFront24:    Re-rank ParetoFront based on crowding distances25:**end procedure**26:**procedure** IterativeRefinement($MBs_{\mathrm{ranked}}$)27:    **while** new data or insights available **do**28:        Update $MBs_{\mathrm{ranked}}$ by adding/removing MBs based on new evaluations29:        Re-apply procedures for identifying Pareto Front and ranking30:    **end while**31:**end procedure**32:ParetoFront←IdentifyParetoFront(MBs)33:SecondaryRanking(ParetoFront)34:ApplyCrowdingDistance(ParetoFront)35:$MBs_{\mathrm{ranked}}\leftarrow$IterativeRefinement(ParetoFront)36:**return** $MBs_{\mathrm{ranked}}$

D-separation is a criterion used to decide whether a set of hyperparameters is conditionally independent of another set, given a third set of hyperparameters, within a Bayesian network. It systematically checks for blocked paths (considering chains and colliders) to determine independence. A path *D* between a hyperparameter $X_{i}$ and hyperparameter $Y_{i}$ is D-separated by a set of hyperparameters *S* if and only if the following:

- *D* includes a chain $X_{i}\leftarrow Z_{i}\to Y_{i}$ such that the middle hyperparameter $Z_{i}$ is in *S*.
- *D* includes a collider $X_{i}\to Z_{i}\leftarrow Y_{i}$ such that the middle hyperparameter $Z_{i}$ is not in *S* and none of $Z_{i}$’s successors are in *S*.

A hyperparameter set *S* is said to D-separate $X_{i}$ and $Y_{i}$ if and only if *S* blocks every path *D* from a hyperparameter $X_{i}$ to a hyperparameter $Y_{i}$. D-separation is indirectly related to the identification of a POMB because it provides a methodological way to verify the conditional independencies within the network. When constructing or analyzing the Markov blanket of a target variable, D-separation can be used to validate whether the selected hyperparameters (forming a potential Markov blanket) indeed render the target variable conditionally independent of all hyperparameters not in the blanket. This validation is essential for ensuring that the identified Markov blanket is minimal and optimal, aligning with the goals of Pareto optimality by not including unnecessary (redundant without adding predictive value) hyperparameters. In achieving a Pareto optimal Markov blanket, one must balance between including relevant hyperparameters (those directly influencing or influenced by the target variable and its spouses via V-structures) and avoiding redundancy (ensuring that the inclusion of any hyperparameter does not unnecessarily duplicate information already captured by the blanket, as can be verified through D-separation).

Pareto optimality emphasizes a balance where no hyperparameter can be added to or removed from the Markov blanket without worsening the balance between relevance (predictive power towards the target variable) and redundancy (overlapping information). D-separation helps ascertain the conditional independencies that justify the exclusion of certain hyperparameters from the Markov blanket, while the understanding of V-structures ensures all relevant direct and indirect (through spouses) influences are considered.

Algorithm 2 outlines a structured procedure to find a POMB for hyperparameter optimization. The algorithm starts by identifying potential Markov blankets for each hyperparameter, considering both direct influences (parents and children) and indirect ones (spouses) found through V-structure detection. Each identified Markov Blanket is then evaluated for its predictive performance and redundancy, using D-separation to ensure that included hyperparameters maintain the target performance metric’s conditional independence. The final step involves ranking these Markov blankets by their balance of predictive performance against redundancy, selecting the top-ranked set as the POMB.
**Algorithm 2** POMB hyperparameter optimization 1:**Input:** Bayesian network B of hyperparameters H and performance metrics P 2:**Output:** Pareto optimal Markov blanket (POMB) for hyperparameters 3:**procedure** IdentifyPOMB(B, H, P) 4:    Initialize POMB←∅ 5:    **for** each hyperparameter $h_{i}\in H$ **do** 6:        Identify $PC(h_{i})$ and $SP(h_{i})$ using V-Structure detection 7:        $MB\left(h_{i}\right)\leftarrow PC\left(h_{i}\right)\cup SP\left(h_{i}\right)$ 8:        Evaluate $MB(h_{i})$ for predictive performance and redundancy 9:    **end for**10:    Rank $MB(h_{i})$ sets by Pareto optimality criteria11:    POMB← Select top-ranked Markov blankets12:    **return** POMB13:**end procedure**14:**procedure** VStructureDetection(B, $h_{i}$)15:    // Detect V-structures involving $h_{i}$16:    Identify child nodes *C* of $h_{i}$17:    **for** each pair $\left(c_{j},c_{k}\right)$ in *C* without a direct link **do**18:        **if** $c_{j}$ and $c_{k}$ have a common child $c_{m}$ **then**19:           Report V-structure $h_{i}\to c_{m}\leftarrow h_{k}$20:        **end if**21:    **end for**22:**end procedure**23:**procedure** EvaluateMarkovBlanket(MB, P)24:    // Evaluate based on D-separation and performance metrics25:    Use D-separation to check conditional independencies within MB26:    Assess predictive performance using P27:    Calculate redundancy score for hyperparameters in MB28:    **return** Combined evaluation score29:**end procedure**

The identification, evaluation, and selection of the POMB are structured around the principles of Bayesian network analysis. Initially, the algorithm employs V-structure detection to meticulously identify potential hyperparameters that directly or indirectly influence the target performance metric, ensuring the inclusion of all relevant and strongly connected hyperparameters. Subsequently, D-separation is utilized to evaluate the conditional independencies among these hyperparameters, refining the initially identified set by removing any hyperparameters that do not contribute to the predictive power or introduce redundancy, thereby ensuring the Markov blanket’s minimality and relevance. The selection of the POMB is then carried out by ranking the refined sets of hyperparameters based on their collective predictiveness and non-redundancy, adhering to Pareto optimality criteria, which systematically balances the trade-off between the complexity of the hyperparameter set and the performance of the model, selecting the optimal set that achieves the best performance without unnecessary complexity. Through these steps, the algorithm navigates the hyperparameter space efficiently, ensuring that the selected POMB is both effective in prediction and efficient in configuration.

#### 3.4.6. Refinement and Validation of Markov Blanket

Algorithm 3 outlines a procedure that explicitly utilizes V-structure detection and D-separation to refine and validate the Markov blanket. The process starts with an initial Markov blanket and refines it by ensuring all relevant hyperparameters involved in V-structures pointing to the target variable are included, and those not contributing to such structures or validated dependencies via D-separation are reconsidered for exclusion. This refinement and validation step is crucial for ensuring that the final Markov blanket accurately captures the essential hyperparameters that influence the target variable’s performance, adhering to both the structural integrity of the Bayesian network and the underlying data-driven relationships.
**Algorithm 3** Refinement and validation of Markov blanket using V-structure detection and D-separation 1:**procedure** RefineAndValidateMB(B, MB(T)) 2:    **Input:** Bayesian network B, initial Markov blanket MB(T) for target *T* 3:    **Output:** Refined and validated Markov blanket $MB_{\mathrm{refined}}\left(T\right)$ 4:    $MB_{\mathrm{refined}}\left(T\right)\leftarrow MB\left(T\right)$                                                                                               ▹ Refine MB using V-structure detection 5:    **for** each hyperparameter $h_{i}$ in $MB_{\mathrm{refined}}\left(T\right)$ **do** 6:        **if** $h_{i}$ is part of a V-structure pointing to *T* **then** 7:           Ensure $h_{i}$ and its spouses are included in $MB_{\mathrm{refined}}\left(T\right)$ 8:        **else** 9:           Remove $h_{i}$ from $MB_{\mathrm{refined}}\left(T\right)$ if it only forms V-structures not pointing to *T*10:        **end if**11:    **end for**                                                                                                      ▹ Validate MB using D-separation12:    **for** each pair of hyperparameters $\left(h_{i},h_{j}\right)$ in $MB_{\mathrm{refined}}\left(T\right)$ **do**13:        Identify all paths *P* between $h_{i}$ and $h_{j}$14:        **for** each path *p* in *P* **do**15:           **if** path *p* is D-separated by $MB_{\mathrm{refined}}\left(T\right)\setminus \left\{h_{i},h_{j}\right\}$ **then**16:               Path *p* does not introduce dependency; continue17:           **else**18:               Path *p* introduces dependency; refine $MB_{\mathrm{refined}}\left(T\right)$ accordingly19:           **end if**20:        **end for**21:    **end for**22:    **return** $MB_{\mathrm{refined}}\left(T\right)$23:**end procedure**

Such V-structure detection helps identify cases where two hyperparameters independently influence a third variable (often a performance metric or another hyperparameter), which can signify a critical interaction that should be preserved in the optimization process. Our approach ensures that hyperparameters involved in V-structures are included in the POMB, as the algorithm acknowledges the importance of these conditional dependencies in predicting the target variable, and this helps with the inclusion of hyperparameters that might otherwise be overlooked if only direct dependencies were considered, thereby enhancing the model’s predictive performance by capturing more nuanced interactions within the network.

Confirming D-separation between hyperparameters serves to refine the set of optimal hyperparameters by verifying conditional independencies. If a set of hyperparameters is D-separated from the target variable given another set of hyperparameters, this indicates that the former set does not directly influence the target when the latter set’s information is available. Thus, hyperparameters that do not contribute additional predictive power or are conditionally independent of the target variable—given the rest of the selected hyperparameters—can be deemed redundant and excluded from the POMB, which reduces the complexity of the hyperparameter set, ensuring that only the most relevant and nonredundant hyperparameters are retained, which simplifies the model and potentially improves generalization by avoiding overfitting.

### 3.5. Evaluation Metrics

#### 3.5.1. Evaluation of Image Enhancement Results

In our experiments to measure the performance of the models, we used SSIM (structural similarity index measure), PSNR (peak signal-to-noise ratio) and LPIPS (learned perceptual image patch similarity).

Peak signal-to-noise ratio (PSNR) is a image quality metric, which measures difference in decibels between pixel intensity values. Higher metric value indicates better image quality. However, metric does not reflect perceptual image quality. Metric is defined in Equation (19).

(19) $$PSNR=10\log_{10}\left(\frac{255^{2}}{MSE}\right),$$

where *MSE* is the mean squared error or L2 loss defined in Equation (20).

(20) $$MSE=\frac{1}{m*n}\sum_{i=0}^{m-1}\sum_{j=0}^{n-1}{\left[I(i,j)-K(i,j)\right]}^{2},$$

where an *m* × *n* sized image *I* is approximated by image *K*, and *i, j* are counters for each image dimension.

Structural similarity index measure (SSIM) is another image quality metric, which focuses on visible structure distortions in the image in three channels: luminance, contrast, and structure, which are measured from mean, standard deviation, and cross-covariance between two images. Metric higher value means images are less different. However, metric as well as PSNR are only considering pixel intensities, which means this metric is not capable to capture perceptual quality. Equation of SSIM is noted in Equation (21), the luminance term in Equation (22), the contrast term in Equation (23), and the structure term in Equation (24).

(21) SSIM(x,y)=l(x,y)c(x,y)s(x,y),

(22) $$l\left(x,y\right)=\frac{2\mu_{x}\mu_{y}+C_{1}}{\mu_{x}^{2}+\mu_{y}^{2}+C_{1}},$$

(23) $$c\left(x,y\right)=\frac{2\sigma_{x}\sigma_{y}+C_{2}}{\sigma_{x}^{2}+\sigma_{y}^{2}+C_{2}},$$

(24) $$s\left(x,y\right)=\frac{\sigma_{xy}+C_{3}}{\sigma_{x}\sigma_{y}+C_{3}},$$

where μ is the mean, σ is the standard deviation, and $\sigma_{xy}$ is the cross-covariance of images *x* and *y*.

Learned perceptual image patch similarity (LPIPS) is a perceptual image quality metric defined in [69]. It is an extension of feature reconstruction loss first described in [70,71]. The difference between the two is that feature reconstruction loss calculates Euclidean distance, whereas LPIPS calculates the MSE distance between feature maps extracted from two images. Another difference is that LPIPS extracts features from multiple layers, whereas feature reconstruction loss uses only one-layer activations. Feature maps are extracted from layers deeper in the model [72], which capture finer details of the images. Originally, VGG-19 was used to retrieve the features, where the model would be trained on ImageNet [73] dataset. LPIPS metric is defined in Equation (25).

(25) $$LPIPS\left(x,y\right)=\frac{1}{m}\sum_{j=1}^{m}MSE\left(\phi_{j}{\left(x\right)}_{h,w,c},\phi_{j}{\left(y\right)}_{h,w,c}\right),$$

where *m* is a number of layers, *j* is a layer index, *x* is a generated image, *y* is a target image, *j* is a convolution layer, ϕ is a feature map, and *h*, *w*, *c* are image height, width and channel dimensions.

#### 3.5.2. Evaluation of Detection of MCI Task

To evaluate models’ performance on detection of MCI task, we utilized widely used metrics such as specificity, sensitivity, and accuracy. Metrics are briefly described in Table 4.

## 4. Results

### 4.1. Preparation of Datasets Used for Detection of MCI

**Figure 2:**
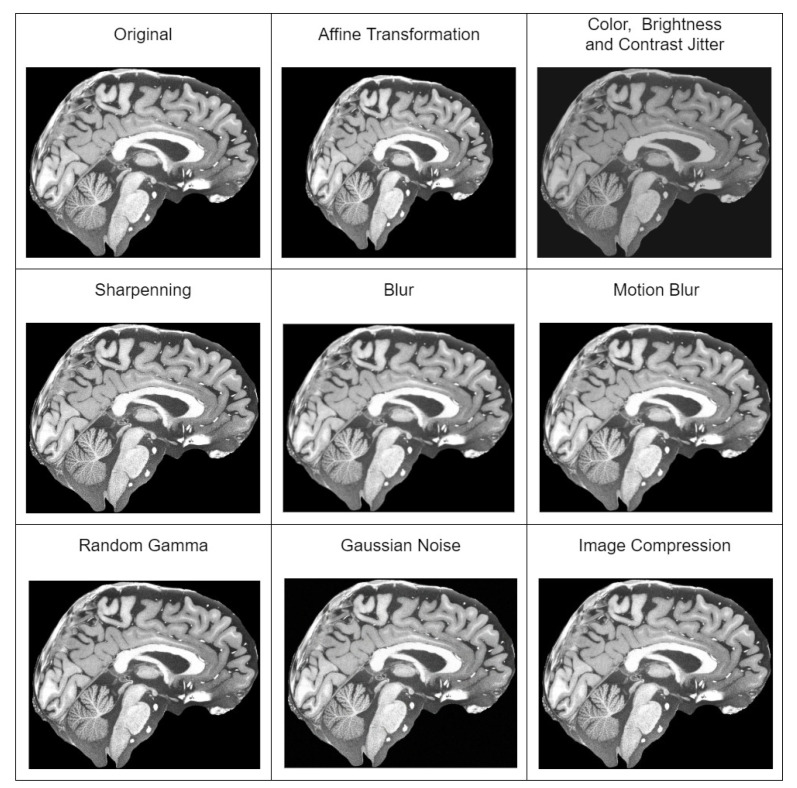
All different augmentation techniques used during training of detection of MCI model. The slice of the brain in this figure is taken from T1w MRI of a healthy 39-year-old male from “human phantom” dataset [52].

**Figure 3:**
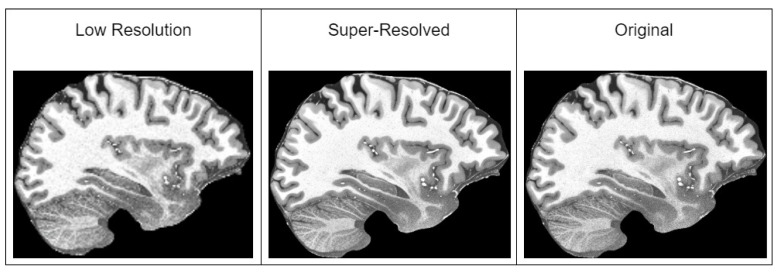
Example of super-resolved low-resolution image with our improved method. The slice of the brain in this figure is taken from T1w MRI of a healthy 39-year-old male from “human phantom” dataset [52].

For the validation of the methodology in the detection of the MCI task, we used ADNI (Alzheimer’s Disease Neuroimaging Initiative) [50] and the Open Access Series of Imaging Studies (OASIS) v4 [74] datasets. We combined both datasets to have a broader spectrum of images in our training and validation sets, and we prepared three datasets out of the combined full dataset. Initially, all datasets were preprocessed with our suggested MRI preprocessing pipeline [25], which included spatial normalization, intensity normalization, and skull stripping. Then, we extracted mid slices (sagittal, coronal and axial) of the brain from each patient, which were resized to 256 × 256 resolution. Dataset descriptions are given below:

1. Only preprocessed with the standard pipeline.
2. Additionally using augmentation techniques—affine transformation, color, brightness and contrast jitter, sharpening, blur and motion blur, Gaussian noise, gamma, and image compression transformations. All of the augmentation techniques used are depicted in Figure 2.
3. Additional to augmentations, before applying augmentation, it super-resolves the preprocessed slices to 1024 × 1024 resolution with the improved super-resolution method. An example of a super-resolved image is depicted in Figure 3.

Each dataset was split in training and validation sets with a proportion of 80/20. Since we only used three slice images of the brain in each plane (sagittal, coronal, axial) for each patient, there was no risk of data leakage. The same patient slices cannot appear in training and in validation.

### 4.2. Models Used in Detection of MCI

For the model architectures to use in the detection of MCI, we chose some of the state-of-the-art models that are not vision transformers due to the fact that transformers are very resource-hungry. Therefore, all selected models were either based on dense or convolution layers. The evaluated model architectures are listed in Table 5.

### 4.3. Implementation Details

The training environment is a personal computer with an AMD Ryzen 5900X CPU, RTX 4090 GPU and 32GB RAM.

The super-resolution model was trained with the batch size of 4, cosine annealing learning rate scheduler, 600 k iterations with a minimum learning rate of 1 × 10^−7^. The starting learning rate was equal to 1 × 10^−4^. For the optimizer, we used Adam with a weight decay of 1 × 10^−3^.

The classification model was trained with a batch size of 32, cross-entropy loss for 600 epochs, and an Adam optimizer with fixed learning rate of 2 × 10^−5^.

### 4.4. Results and Discussion of Improved Super-Resolution Method

All of the results that we captured during validation of trained models with different discriminators are listed in Table 6.

**Figure 4:**
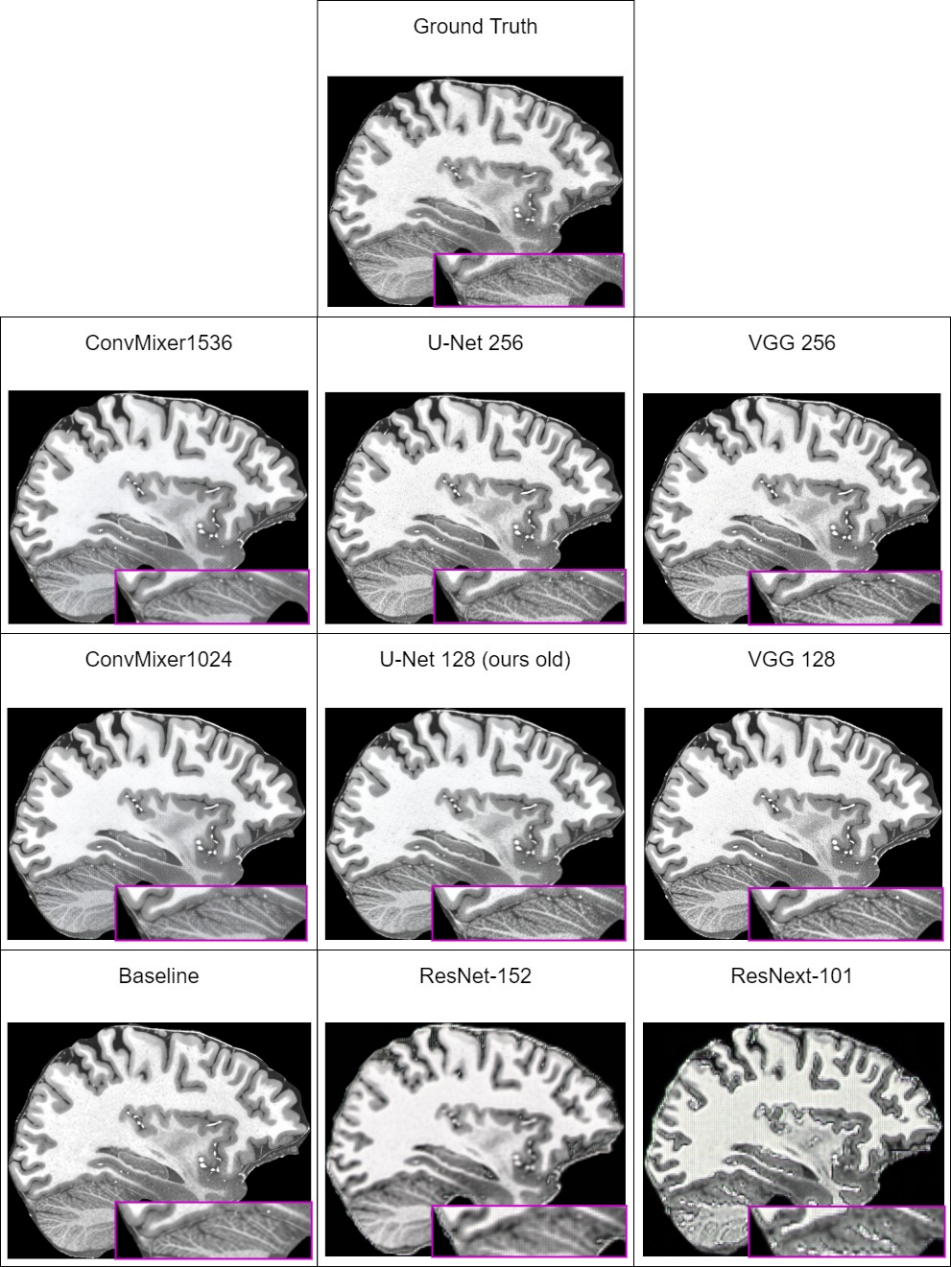
Subjective comparison of super-resolved low-resolution images with our improved method. The ground truth slice of the brain in this figure is taken from MPRAGE T1w MRI that was taken with Siemens 7T Classic MR scanner from “human phantom” dataset [52]. Purple area shows zoomed in section of the brain to better visualize differences between models.

In Table 6, we can see that the best perceptual quality results are achieved with the ConvMixer1536 model used as discriminator. However, looking at the subjective comparison in Figure 4, it seems that the LPIPS metric does not capture artifacts that are present in images generated by ConvMixer models. Comparing subjectively generated images, images generated using U-Net or VGG are far more close to ground-truth images. This means that LPIPS is unable to correctly quantify perceptual quality of generated images. Similar remarks were made by other researchers, for example, those in [78] (which investigated why artifacts appear and how to reduce them) that all currently used perceptual quality metrics are unable to capture existence of these artifacts in the generated images as a decrease in the metric score.

Excluding the fact that LPIPS does not capture artifacts, and therefore, results with ConvMixers are not subjectively best, new methodology improvements increased all of the metric values over the last iteration. The best overall result is achieved with the U-Net discriminator, which uses 256 input features.

### 4.5. Results and Discussion of Detection of MCI Task

**Figure 5:**
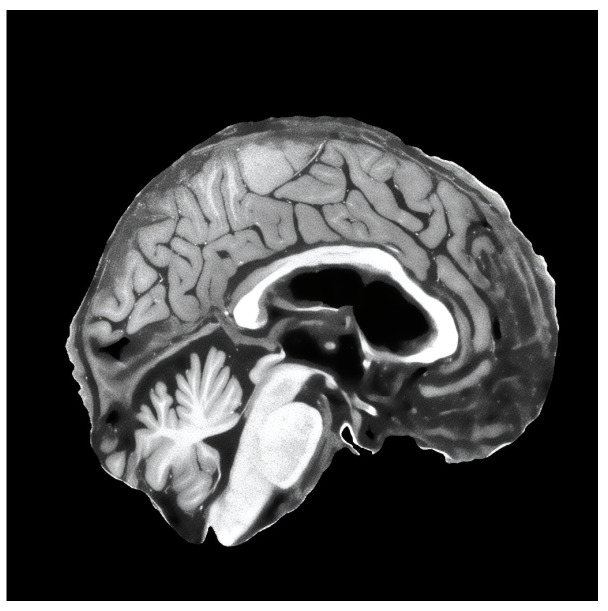
Example of a generated brain image of sagittal plane from ADNI [50] dataset, which contains black spots. The slice of the brain in this figure is taken from MPRAGE T1w MRI, which was taken with 3T MR scanner.

Preparing a third dataset required us to use our new methodology to upscale images into 1024 × 1024 resolution. Initial upscaling finding showed us that we faced a domain shift problem, where our developed model performed poorly on a different dataset used in training. We used the ultra-high-resolution MRI dataset “human phantom” [52]. Our model subjectively was generating good results on the OASIS-4 dataset, but when we tried to run it against ADNI dataset, we found that generated images in some cases contain what we could call “black spot” artifacts Figure 5. This is a typical generalization problem, when the dataset used in real-life usually differs from the one used during training. The best solution in our case is to expose the model to the new data during training using fine-tuning—taking the already-trained model and re-training it with the new data added to the dataset.

The first step was to upscale all ADNI dataset images and then manually pick those that did not contain “black spot” artifacts, then add those images to the original dataset and fine-tune the already-trained model. After training, the model was able to generate images without “black spot” artifacts.

The second step was to train MCI detection models with three prepared datasets. Validation results are listed in Table 7.

Across a majority of trained models, there were big differences between sensitivity and specificity metrics, which means that models tended to overfit the data. However, in the sagittal and coronal planes, ConvMixer reached the best overall accuracy in the detection of MCI. In the axial plane, the best model was EfficientNet.

The next step was to validate the models against dataset with augmentation techniques. The results are listed in Table 8.

The overall improvement using augmentation was on average around 5%. Here again, ConvMixer showed a lead in the sagittal and coronal planes, whereas on the axial plane, it fell shortly behind AlexNet. The last step to verify the effect of super-resolution on the detection of MCI was to validate models on the third dataset, which used super-resolution and all the augmentation techniques that the second dataset used. The validation results are listed in Table 9.

Comparing results between the second dataset and third, it is obvious that the super-resolution methodology has improved the stability of models, because all models show a small difference between sensitivity and specificity. Additionally, all models across the table show performance improvements of 1–8%, on average 4%, which means that our proposed methodology has a positive effect on the performance of models in the MCI detection task. What is interesting is that in the sagittal and coronal planes with super-resolution, ResNet is showing the best results. This may be due to the fact that the third dataset is using higher-quality images, which yields more features, and it is possible that ResNet residual connections allow the model to retain more important features that are contributing to the accuracy of prediction.

## 5. Discussion and Conclusions

This study introduces a novel advancement in the detection of mild cognitive impairment (MCI) by applying super-resolution techniques to structural MRI images and optimizing deep learning models using a Pareto optimal Markov blanket (POMB). This approach notably enhances the perceptual quality of MRI images, which subsequently improves the accuracy of various state-of-the-art classifiers in identifying MCI. An improvement in detection accuracy ranging from 1–4% was observed, underscoring the efficacy of super-resolution in enhancing diagnostic models.

The incorporation of a POMB for hyperparameter optimization emerges as a key innovation, streamlining the exploration of complex hyperparameter spaces by focusing on parameters that impact the target variable, either directly or indirectly. This strategy not only accelerates the optimization process but also significantly mitigates the risk of overfitting by ensuring a balance between model complexity and performance. As a result, models demonstrate robustness and generalizability across different datasets, a critical advantage in medical diagnostics.

An important insight from this research is the impact of discriminator choice in generative adversarial network (GAN) setups on the perceptual quality of super-resolved images. The study’s comparison reveals that discriminators like VGG and U-Net produce significantly different outcomes, with U-Net marginally superior in PSNR and SSIM metrics. This highlights the profound influence of discriminator selection on both subjective and objective image quality.

A notable discovery pertains to the limitations of the learned perceptual image patch similarity (LPIPS) metric. Despite indicating high perceptual quality for images generated by ConvMixer models, subjective assessments contradicted these findings, revealing poor quality. This discrepancy suggests a pressing need for a new metric capable of accurately detecting "checkerboard" artifacts and properly quantifying perceptual quality differences.

In conclusion, this study advances the field of medical imaging and MCI detection, demonstrating the potent application of super-resolution processing and the crucial role of hyperparameter optimization and discriminator selection in creating accurate and reliable diagnostic models. The findings advocate for ongoing research into more effective perceptual quality metrics, further enhancing the utility of super-resolution in medical diagnostics.

## Figures and Tables

**Table 2:** Description of datasets used in classification of MCI.

Dataset	Description	# of Samples Used
ADNI	First version released in 2004. Focus on Alzheimer’s disease and its early-stage MCI. We only used T1w MRI images, although it has many other data modalities.	689 MCI, 689 CN
OASIS-4	First version released in 2007. Focus on memory disorders and dementia. We also utilized only T1w MRI images.	47 MCI, 47 CN

CN—Cognitive Normal (Healthy Patient), ADNI—Alzheimer’s Disease Neuroimaging Initiative, OASIS—Open Access Series of Imaging Studies.

**Table 3:** Model architectures used for discriminator in GAN loss.

Model	Reference	Used Permutations of Model
VGG-16	[40]	With 128 and 256 input features.
ConvMixer	[53]	(width, depth, kernel size, patch size): (1536, 20, 9, 7) (1024, 20, 9, 14)
U-Net	[54]	With 128 and 256 features
ResNet-152	[55]	Only original implementation
ResNext-101	[56]	Only original implementation

**Table 4:** Metrics used For detection of MCI task.

Metric	Description	Formula
Accuracy	Sum of number *N* image predictions, where result is 1 if label and prediction match, and 0 otherwise.	(26) $\frac{1}{N}\sum_{i}^{N}1\left(y_{i}={\hat{y}}_{i}\right)$
Specificity	Rate of true negative, which describes the probability that a negative prediction is actually negative.	(27) $\frac{TN}{TN+FP}$
Sensitivity	Rate of true positive, which describes the probability that a positive prediction is actually positive.	(28) $\frac{TP}{TP+FN}$

**Table 5:** Model architectures used for detection of MCI task.

Model	Reference	Variations
ConvMixer	[53]	Width = 1536, Depth = 20, Kernel Size = 9, Patch Size = 7.
ResNet	[55]	152.
AlexNet	[75]	No variations.
EfficientNet	[76]	B7.
DenseNet	[77]	201.

**Table 6:** Objective comparison of models used for discriminator to improve our previous super-resolution HAT model published in [25].

Model	SSIM ↑	PSNR ↑	LPIPS ↓
HAT + ConvMixer1536	88.966	29.621	0.0463
HAT + U-Net 256	88.612	28.809	0.0514
HAT + VGG 256	88.493	28.532	0.0515
HAT + ConvMixer1024	88.695	29.208	0.0519
HAT + U-Net 128 (ours old)	88.585	28.742	0.0529
HAT + VGG 128	88.424	28.366	0.0541
HAT (baseline)	91.406	31.765	0.0984
HAT + ResNet-152	84.460	25.303	0.1189
HAT + ResNext-101	81.170	24.457	0.1883

**Table 7:** Objective comparison of models used for detection of MCI on the first dataset (no augmentation).

Plane	Model	Accuracy	Sensitivity	Specificity
Sagittal	ConvMixer-1536	0.8966	0.8288	0.9641
	AlexNet	0.8876	0.9144	0.8610
	EfficientNet-B7	0.8562	0.8198	0.8923
	ResNet-152	0.8180	0.7117	0.9237
	DenseNet-201	0.7978	0.6261	0.9698
Axial	EfficientNet-B7	0.8899	0.8738	0.9058
	ResNet-152	0.8854	0.8468	0.9238
	AlexNet	0.8539	0.8468	0.8609
	ConvMixer-1536	0.7124	0.5360	0.8878
	DenseNet-201	0.6382	0.3333	0.9417
Coronal	ConvMixer-1536	0.8337	0.7747	0.8923
	ResNet-152	0.8292	0.7072	0.9506
	AlexNet	0.8270	0.8153	0.8385
	EfficientNet-B7	0.8135	0.7027	0.9237
	DenseNet-201	0.7865	0.7387	0.8340

**Table 8:** Objective comparison of models used for detection of MCI on the second dataset (with augmentation).

Plane	Model	Accuracy	Sensitivity	Specificity
Sagittal	ConvMixer-1536	0.9281	0.8783	0.9775
	EfficientNet-B7	0.9281	0.9369	0.9192
	Resnet-152	0.9236	0.9279	0.9192
	DenseNet-201	0.9101	0.9054	0.9147
	AlexNet	0.8809	0.8603	0.9013
Axial	AlexNet	0.9213	0.9279	0.9147
	ConvMixer-1536	0.9146	0.9730	0.8565
	EfficientNet-B7	0.9146	0.9234	0.9058
	DenseNet-201	0.9079	0.8603	0.9551
	ResNet-152	0.8989	0.9189	0.8789
Coronal	ConvMixer-1536	0.9438	0.9414	0.9461
	ResNet-152	0.9416	0.9820	0.9013
	EfficientNet-B7	0.9371	0.9234	0.9506
	DenseNet-201	0.9101	0.9234	0.8968
	AlexNet	0.9079	0.8513	0.9641

**Table 9:** Objective comparison of models used for detection of MCI on the second dataset (with super-resolution and augmentation).

Plane	Model	Accuracy	Sensitivity	Specificity
Sagittal	ResNet-152	0.9371	0.9369	0.9372
	EfficientNet-B7	0.9348	0.9369	0.9327
	ConvMixer-1536	0.9326	0.9459	0.9192
	DenseNet-201	0.9326	0.9369	0.9282
	AlexNet	0.9281	0.9324	0.9237
Axial	EfficientNet-B7	0.9348	0.9549	0.9147
	ConvMixer-1536	0.9326	0.9414	0.9237
	AlexNet	0.9213	0.9099	0.9327
	ResNet-152	0.9213	0.9414	0.9013
	DenseNet-201	0.9191	0.9234	0.9147
Coronal	ResNet-152	0.9573	0.9549	0.9596
	EfficientNet-B7	0.9551	0.9459	0.9641
	ConvMixer-1536	0.9438	0.9414	0.9461
	DenseNet-201	0.9438	0.9324	0.9551
	AlexNet	0.9011	0.8963	0.9058

## Data Availability

We used ADNI (Alzheimer’s Disease Neuroimaging Initiative) [50] and the Open Access Series of Imaging Studies (OASIS) v4 [74] datasets, and the “Human Phantom” dataset, available online at https://datadryad.org/stash/dataset/doi:10.5061/dryad.38s74 (accessed on 5 March 2024).

## Abbreviations

The following abbreviations are used in this manuscript:

EEG	Electroencephalogram
FDG-PET	Fluoro-deoxy-glucose positron emission tomography
CSF	Cerebrospinal fluid
ROI	Regions of interest
POMB	Pareto optimal Markov blanket
SSIM	Structural similarity index measure
DAG	Directed acyclic graphs
GAN	Generative adversarial network
WGAN	Wasserstein GAN
FFT	Fast Fourier transform
ADNI	Alzheimer’s Disease Neuroimaging Initiative
OASIS	Open Access Series of Imaging Studies
PSNR	Peak signal-to-noise ratio
MCI	Mild cognitive impairment
HAT	Hybrid attention transformer
LPIPS	Learned perceptual image patch similarity
HR-MRI-GAN	High-resolution MRI generative adversarial network
CNN	Convolutional neural network
SVM	Support vector machine
